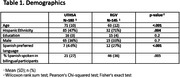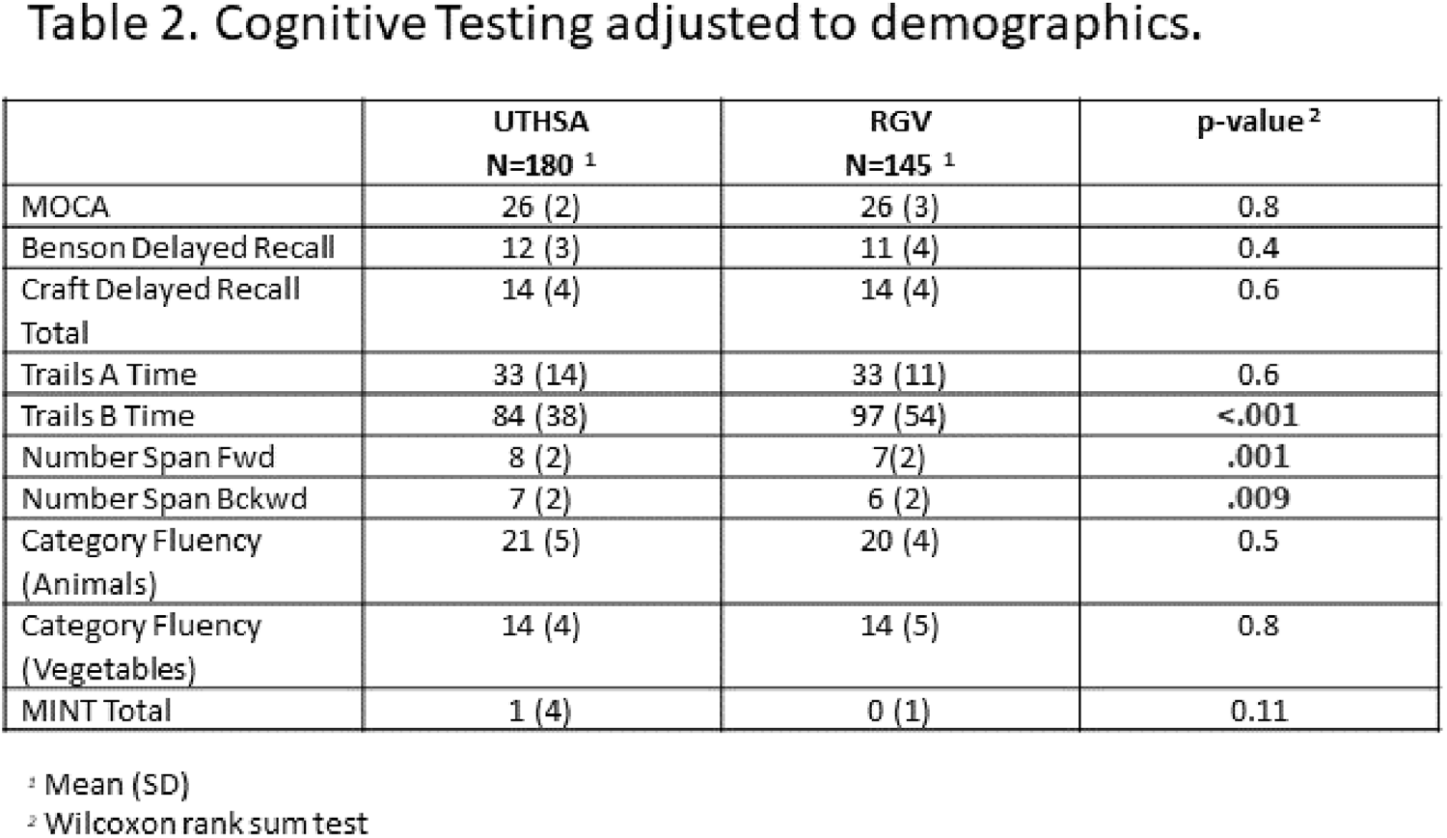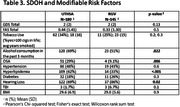# Multi Site Comparison of Participant Profiles in the South Texas Alzheimer’s Disease Research Center

**DOI:** 10.1002/alz.091760

**Published:** 2025-01-03

**Authors:** Jennifer G Del Bosque, Chen‐Pin Wang, Roman A. Fernandez, Jeremy A. Tanner, A. Campbell Sullivan, Sarah E. Savoia, Alicia S. Parker, Arash Salardini, Gabriel A. de Erausquin, Monica Goss, Hector A Trevino, Aishwarya N. Patel, Haritha V. Katragadda, Amaya M Seidl, Amy R. Saklad, Amy Werry, Mitzi M. Gonzales, Rosa P. Pirela‐Mavarez, Silvia Mejia‐Arango, Shannon Lavigne, Gabrielle Hromas, Stephanie Santiago‐Mejias, Claudia L Satizabal, Robin C. Hilsabeck, Gladys E. Maestre, Sudha Seshadri, Ashley LaRoche

**Affiliations:** ^1^ Glenn Biggs Institute for Alzheimer’s & Neurodegenerative Diseases, University of Texas Health Science Center, San Antonio, TX USA; ^2^ Glenn Biggs Institute for Alzheimer’s & Neurodegenerative Diseases, San Antonio, TX USA; ^3^ UT Health San Antonio, San Antonio, TX USA; ^4^ University of Texas Health San Antonio, San Antonio, TX USA; ^5^ Glenn Biggs Institute for Alzheimer’s & Neurodegenerative Diseases, University of Texas Health Science Center, San Antonio, TX USA; ^6^ Glenn Biggs Institute for Alzheimer’s and Neurodegenerative Diseases, University of Texas Health San Antonio, San Antonio, TX USA; ^7^ Department of Neurology, University of Texas Health San Antonio, San Antonio, TX USA; ^8^ Glenn Biggs Institute for Alzheimer’s & Neurodegenerative Diseases, University of Texas Health San Antonio, San Antonio, TX USA; ^9^ Glenn Biggs Institute for Alzheimer’s & Neurodegenerative Diseases, University of Texas Health Sciences Center at San Antonio, San Antonio, TX USA; ^10^ Glenn Biggs Institute for Alzheimer’s and Neurodegenerative Diseases, UT Health San Antonio, San Antonio, TX USA; ^11^ Cedars‐Sinai Medical Center, Los Angeles, CA USA; ^12^ The University of Texas Rio Grande Valley School of Medicine, Harlingen, TX USA; ^13^ Glenn Biggs Institute for Alzheimer’s & Neurodegenerative Diseases, University of Texas Health Science Center at San Antonio, San Antonio, TX USA; ^14^ The University of Texas Rio Grande Valley School of Medicine, Brownsville, TX USA; ^15^ Department of Neurology, University of Texas Health Sciences Center, San Antonio, TX USA

## Abstract

**Background:**

Hispanic adults comprise 19% of the US population, yet <8% of Alzheimer’s Disease and Related Dementia (ADRD) research cohorts. Hispanic adults in the US are 1.5 times more likely to develop ADRDs compared to Non‐Hispanics. Considering the projected growth in Hispanics in the US, a >400% increase in ADRDs in this population is anticipated over the next twenty‐five years. Knowledge is limited on the prevalence and influence of social determinants of health (SDoH) and modifiable risk factors on cognition in ethnically diverse groups. The South Texas Alzheimer’s Disease Research Center (STAC, *P30AG066546)* is a collaboration between UT Health San Antonio (UTHSA) and UT Rio Grande Valley (UTRGV) with a primary aim to better understand ADRDs in Hispanic adults. Our objective is (1) to describe differences in SDoH, modifiable risk factors, and cognitive performance in cognitively unimpaired adults in STAC, and (2) to assess differences between sites to better understand the risk factors in ADRDs in Hispanics by location.

**Method:**

Participants in San Antonio, Tx, and Rio Grande Valley, Tx completed cognitive testing and questionnaires of medical history, SDoH, and modifiable ADRD risk factors. The two sites were compared between sites with Wilcoxon and T‐Tests. Differences in neurocognitive performance was further assessed with linear regression adjusting for age, sex, and education.

**Result:**

Cognitively unimpaired adults were enrolled at UTHSA (n = 180, mean(SD) age 71(10), 36% male, 47% Hispanic) and UTRGV (n = 45, age 63(12), 33% male, 71% Hispanic). Participants at UTRGV were younger and more frequently identified as Hispanic. Site differences were seen on Trails B (p < 0.001), and Number Span Total Forward (p = .001) and Total Backward (p = 0.009) after adjusting for age, sex, and education. Sites showed differences in risk factors including hyperlipidemia (p = <.001), hearing loss (p = .022), Sleep Apnea (p = .006), and alcohol use (p = .022). Other risk factors were similar across sites.

**Conclusion:**

Overall, there were significant differences in the risk factors between locations, supporting the value of recruiting participants at both sites to assess the spectrum of risk factors and SDoH in Hispanic adults.